# A technique for constructing diverting loop ileostomy to prevent outlet obstruction after rectal resection and total colectomy: a retrospective single-center study

**DOI:** 10.1007/s00595-021-02381-8

**Published:** 2021-10-24

**Authors:** Yusuke Takehara, Mihoko Nakagawa, Hiroaki Kobayashi, Kensuke Kakisako, Yojiro Takano, Junichi Seki, Shoji Shimada, Kenta Nakahara, Shumpei Mukai, Yuta Enami, Naruhiko Sawada, Fumio Ishida, Shin-ei Kudo

**Affiliations:** Digestive Disease Center, Showa University Northern Yokohama Hospital, 35-1 Chigasaki-chuo, Tsuzuki-ku, Yokohama, Kanagawa Prefecture 224-8503 Japan

**Keywords:** Outlet obstruction, Diverting loop ileostomy, Oral superior

## Abstract

**Purpose:**

Preventing outlet obstruction associated with a diverting stoma is important. Previously, we constructed a diverting loop ileostomy with the proximal limb of the small intestine on the caudal side, namely the oral inferior (OI) method. However, to address the issue of twisting and stenosis of the small intestine, we recently constructed a diverting loop ileostomy with the proximal limb on the cranial side, namely the oral superior (OS) method. We compared the incidence of outlet obstruction between the two methods.

**Methods:**

The subjects of this retrospective study were 133 patients who underwent colorectal resection or total colectomy, with D2 or more lymph node dissection and diverting loop ileostomy construction, between April, 2001 and December, 2018, at our hospital. The OI method was performed in 54 patients and the OS method was performed in 79 patients.

**Results:**

In the OS group, a history of laparotomy, neoadjuvant therapy, clinical stage III, and the use of anti-adhesion materials were more common, whereas blood loss and the incidence of outlet obstruction were significantly lower. Multivariate analysis identified only OS placement as a significant factor for reducing the incidence of outlet obstruction.

**Conclusion:**

When constructing a diverting loop ileostomy, placing the proximal limb on the cranial side is important.

## Introduction

Recent remarkable advances in preoperative chemoradiation therapy and surgical techniques for lower rectal cancer and the widespread use of this treatment combination have led to an increase in the number of cases of diverting stoma construction to reduce the risk of postoperative leakage [1, 2]. However, no consensus exists regarding whether ileostomy or colostomy is better [3–6]. In Japan, ileostomy is often selected because it is easy to construct and close [7]. Stoma-related complications include skin disorders, stoma necrosis, stoma prolapse, high-output, parastoma hernia, and bowel obstruction [8–10]. In particular, bowel obstruction, called outlet obstruction (OO), tends to occur with loop ileostomy. Differentiating between OO and bowel obstruction/ileus is difficult because both cause bloating and vomiting, and a diagnosis of bowel obstruction may include OO. However, ileus and OO are separate pathological entities with different causes because ileus may present as diffuse dilation down to the stoma site, whereas OO involves an obstruction at the stoma site. OO may interfere with early meal initiation and necessitate early stoma closure. OO was initially reported as ileostomy with ileus after surgery for ulcerative colitis (UC) or familial adenomatous polyposis (FAP) [11–13], but the number of reports of OO has increased in recent years [11–17]. Several reports on the prevention of OO indicate that a fascial incision should be made vertically rather than by a cross incision to reduce adhesion and twisting of the mesentery, and to create a stoma tunnel with a sufficient margin [8–10, 18, 19]. Furthermore, laparoscopic surgery has been reported as a risk factor for OO [20] and has not yet been considered for preventing OO [8–10, 12, 19, 20]. However, unlike other complications associated with a stoma, the incidence of OO is expected to be reduced by revising the procedure during stoma construction. Therefore, prevention strategies are important when constructing a diverting loop ileostomy (DLI) [9, 12, 19]. Several reports indicate that rotating the proximal limb of the small intestine to the caudal side is better when constructing loop ileostomy; however, it is unclear if rotation of the small intestine can prevent OO [18, 19, 21–23]. In the past, when constructing a DLI in our department, the oral side was the caudal side (oral inferior; OI). However, now we construct it so that the oral side is the cranial side (oral superior; OS) because of recent findings of twist and stenosis of the small intestinal limb. We conducted this retrospective study to examine the incidence of OO after the OI method versus the OS method.

## Methods

**Fig. 1:**
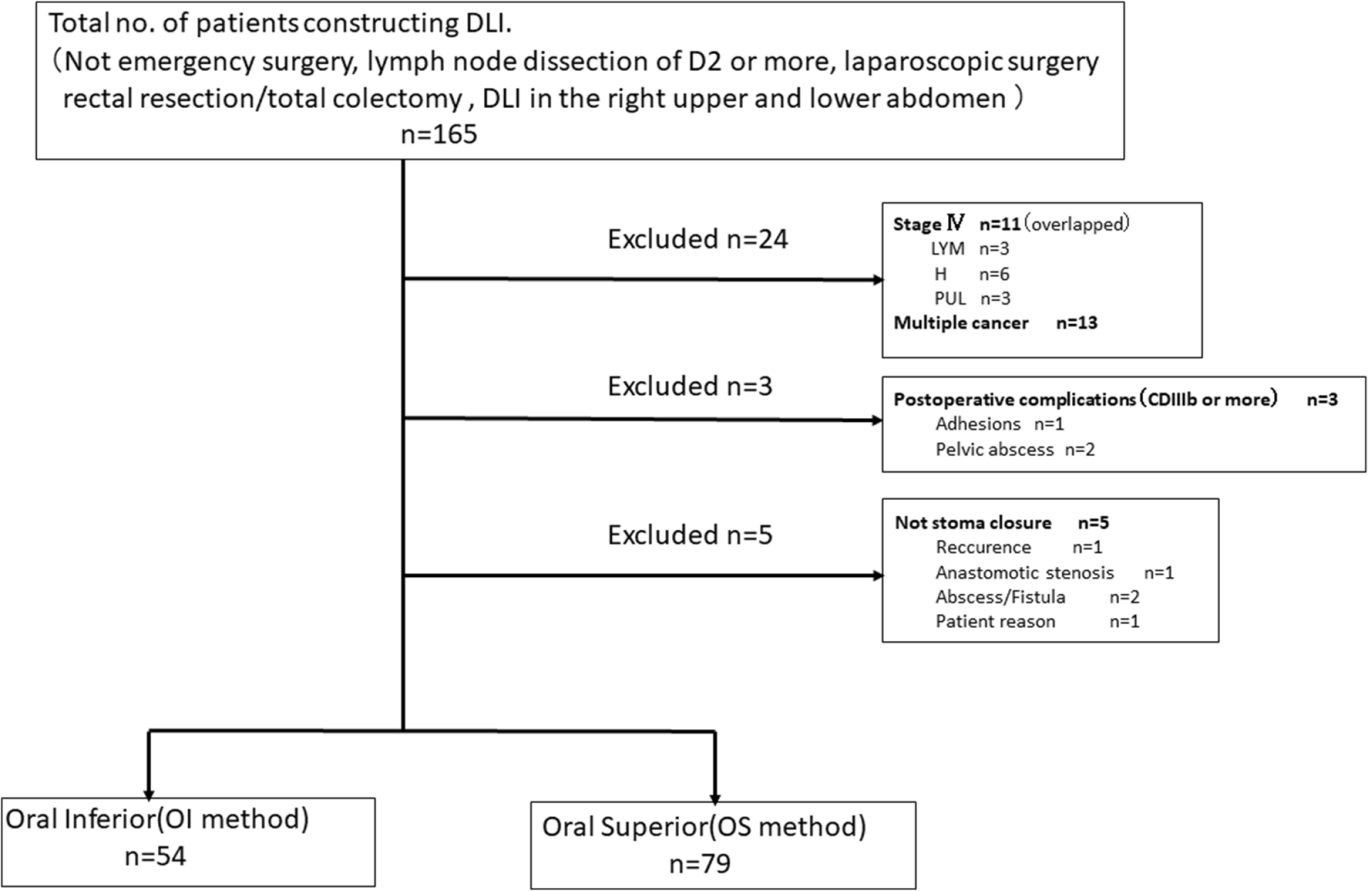
The CONSORT diagram for this study. *CONSORT* Consolidated Standards of Reporting Trials

Between April, 2001 and December, 2018, 165 patients underwent rectal resection or total colectomy with lymph node dissection of D2 or more, with intraoperatively constructed DLI in the right upper or lower abdomen performed laparoscopically. After excluding patients with bowel obstruction caused by colorectal cancer before surgery; those who underwent emergency surgery for reasons such as peritonitis; those with distant cancer metastasis, multiple cancers, or multiple primary cancer; and those with postoperative complications of Clavien–Dindo IIIb or more, 133 patients who had undergone stoma closure were the subjects of this analysis [24] (Fig. 1). There were 54 patients in the OI group and 79 patients in the OS group. The procedure for constructing a DLI in our department can be summarized as follows:

The stoma site was marked before surgery, based on the principles of the Cleveland Clinic (Cleveland, OH, USA). After making a 3- to 4-cm longitudinal skin incision with the apex marked, the rectus abdominis sheath was also incised vertically to the same length. The rectus abdominis muscle was split bluntly to form a stoma tunnel that allowed two lateral fingers to pass sufficiently. After pulling out the small intestine on the oral side, approximately 30–40 cm from the terminal ileum, based on the Brooke method, a loop ileostomy was constructed with approximately 8–10 strands of absorbable suture material on needles, to a height of approximately 5 mm on the anal limb and approximately 30 mm on the proximal limb. Laparoscopically, the ileum was followed from the terminal ileum to confirm that the small intestine on the oral side, approximately 30–40 cm from the terminal ileum, could be elevated to the marked stoma site. To prevent pneumoperitoneum, after returning the patient to the supine position, we constructed the loop ileostomy using the aforementioned method, confirming it directly through a small laparotomy wound. At the conclusion of open and laparoscopic surgery, the main abdominal wound was closed and dressings were applied to exclude it from contamination when the loop ileostomy was opened.

We compared the following factors between the two groups: sex; age; history of laparotomy; body mass index; primary disease (colorectal cancer: neuroendocrine tumor/UC/FAP); neoadjuvant therapy; clinical stage (using the criteria of *UICC TNM Classification of Malignant Tumours*, 8th edition [25]; stage 0–II:III); performance status of 0–1:2–4; surgical procedure, such as rectal resection or total colectomy such as ileal pouch anal anastomosis (IPAA); whether anti-adhesion material such as Seprafilm [Baxter International Inc., Deerfield, IL, USA]) was used; operation time; blood loss; postoperative length of stay in hospital; period until stoma closure; OO; stoma-related complications, excluding bowel obstruction/ileus and OO; the degree of intra-abdominal adhesion; rectus abdominis muscle thickness (horizontal and vertical); length of the straight line connecting both ends of the rectus abdominis muscle; the position where the stoma limbs penetrated the rectus abdominis muscle (center-inside or outer); and the angle between the stoma limbs and the rectus abdominis muscle.

The features of OO are as follows: (1) symptoms of intestinal obstruction such as bloating and vomiting; (2) relief of the bowel obstruction by inserting a decompression catheter trans-stomally; and (3) computed tomography (CT) image of a caliber change in the abdominal wall-penetrating part of the stoma but no other obstruction mechanism. Criteria 1 and 2 or 1 and 3 were satisfied during the period from construction of the DLI to stoma closure.

**Fig. 2:**
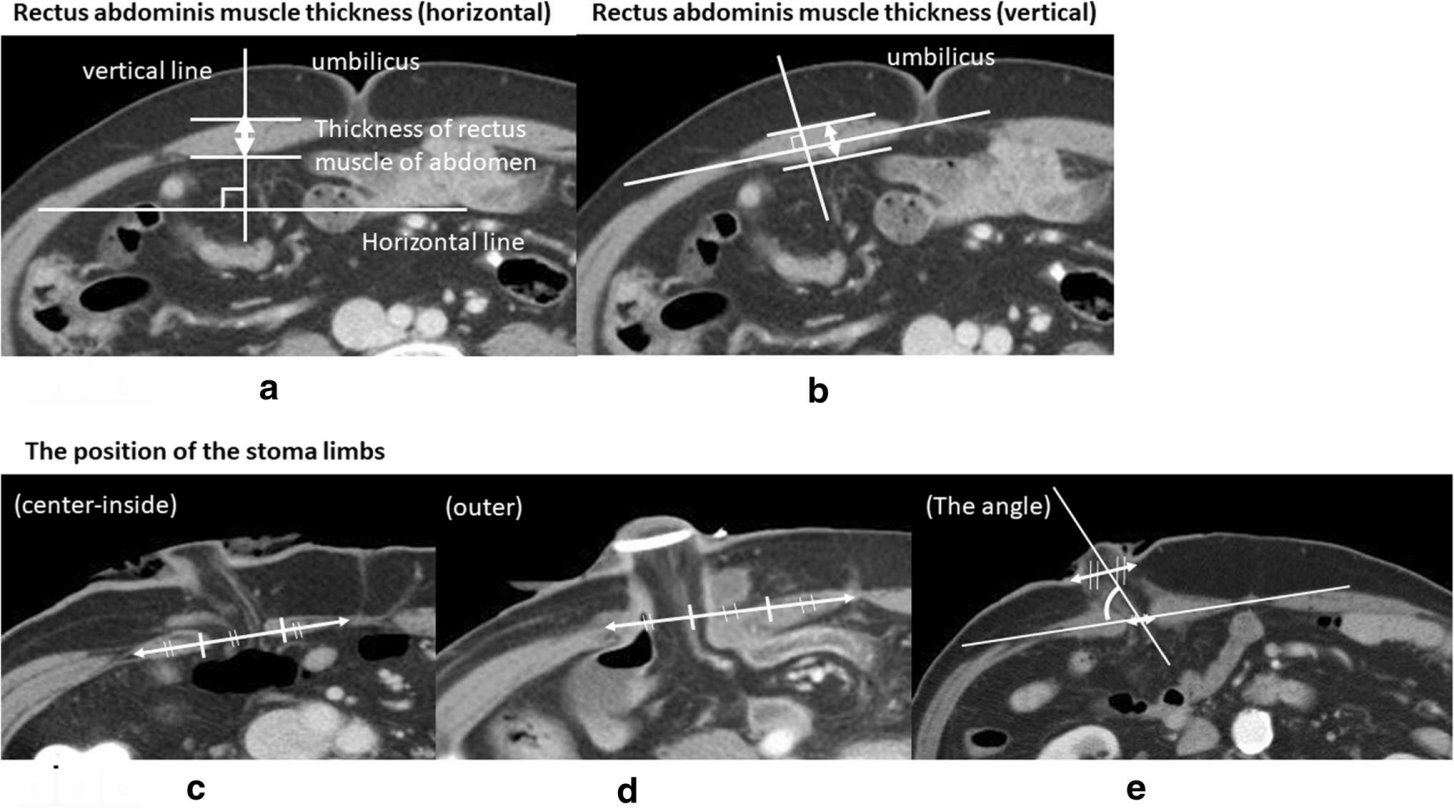
**a** A straight line is drawn perpendicular to the horizontal axis at the thickest part of the right rectus abdominis muscle. **b** A straight line is drawn perpendicular to the straight line connecting both ends of the rectus abdominis muscle. **c** The stoma limbs are classified as penetrating the center-inside. **d** The stoma limbs are classified as penetrating the outside. **e** The angle formed by the straight line connecting both ends of the rectus abdominis muscle and the long axis of the stoma limbs is measured on a CT scan image and on the slice in which the stoma limbs penetrate the rectus abdominis

The horizontal thickness (mm) of the rectus abdominis muscle was measured by selecting an umbilical level slice in the preoperative CT examination and drawing a straight line perpendicular to the horizontal axis at the thickest part of the right rectus abdominis muscle in the image [26] (Fig. 2a). The vertical thickness (mm) of the rectus abdominis muscle was measured by selecting an umbilical level slice in the preoperative CT examination and drawing a straight line perpendicular to the straight line connecting both ends of the rectus abdominis muscle [27] (Fig. 2b). The thickness was classified as < 10 mm or ≥ 10 mm. The length of the straight line connecting both ends of the rectus abdominis muscle was measured by selecting an umbilical level slice in the preoperative CT examination.

The position where the stoma limbs penetrate the rectus abdominis muscle was identified by CT examination before stoma closure in the 64 patients who underwent CT examination before stoma closure. On dividing the straight line connecting both ends of the rectus abdominis into three equal parts, the stoma limbs were classified as penetrating the center-inside (Fig. 2c) or the outside [27] (Fig. 2d). The angle between the stoma limbs and the rectus abdominis was calculated, based on CT scan images before stoma closure. The slice in which the stoma limbs penetrated the rectus abdominis was selected. The angle formed by the straight line connecting both ends of the rectus abdominis muscle and the long axis of the stoma limbs was measured [27] (Fig. 2e). The long axis of the stoma limbs was defined as the line connecting the centers of both short axes, where the stoma limbs penetrate the rectus abdominis muscle and the epidermal level [27] (Fig. 2e).

We evaluated the incidence, extent, and type of adhesions in the abdominal cavity of adhesions during the second laparotomy for stoma closure. A circular laparotomy wound was made around the stoma, which revealed adhesions in the abdominal cavity. Existing adhesions around the midline incision and peristomal area were evaluated as “none” for no adhesions; “mild,” for adhesions covering up to 25% of the total area and length with a filmy thickness and avascularity; “moderate,” for adhesions covering 26%–50% of the total area and length, moderate thickness, and limited vascularity; and “severe,” for adhesions covering > 51% of the total area and length with dense thickness and vascularization. We examined no–mild adhesions and moderate–severe adhesions [28].

Statistical analyses were conducted using JMP Pro 14 software (SAS Institute, Cary, NC, USA). Comparisons were made using Fisher’s exact test, the Chi-squared test, or the *t* test, as appropriate. Multivariate logistic regression analyses were conducted to identify the independent risk factors for OO. Odds ratios and 95% confidence intervals were also estimated. In univariate and multivariate analyses, a value of *p* < 0.05 was considered significant.

Appropriate research ethics and review board permissions were obtained from Showa University Northern Yokohama Hospital (Yokohama, Japan; approval number, 19H093). Informed consent was obtained from the patients who were the subjects of this study, and who were given the choice to opt out.

## Results

Table 1 summarizes the patients’ clinical characteristics. A history of laparotomy, neoadjuvant therapy, and clinical stage (stage III) were significantly more frequent in the OS group, but other factors did not differ significantly between the OI and OS groups. The use of anti-adhesion material was significantly greater and blood loss was significantly less in the OS group (Table 2). There were no significant differences in operation time, postoperative hospital stay, or period until stoma closure, between the groups (Table 2). The incidence of OO was significantly lower in the OS group (Table 3). There were no significant differences in stoma-related complications, except for bowel obstruction/ileus and OO, or in the degree of intra-abdominal adhesions confirmed during stoma closure, between the groups (Table 3). There were no significant differences in the horizontal or vertical thickness of the rectus abdominis muscle (Fig. 2a, b), or in the length of the straight line connecting both ends of the rectus abdominis muscle, or the position where the stoma limb penetrated the rectus abdominis muscle, in the 64 patients who underwent CT examination before stoma closure (Fig. 2c, d) (Table 3). The angle between the stoma limbs and the rectus abdominis (Fig. 2e) was significantly higher in the OS group (Table 3). Multivariate analysis using logistic regression analysis identified that only the OS position was a significant factor in reducing the incidence of OO (Table 4).

**Table 1:** The patients’ characteristics

	OI (*n*=54)	OS (*n*=79)	*P* value
Sex(M: F)	41:13	57:22	0.6914
Age(years)※	63 (32–84)	61.0 (34–84)	0.6577
Past history of laparotomy	11 (20.4%)	21 (26.6%)	0.0047
BMI(kg/m^2^)※	22.9 (15.6–28.8)	22.6 (15.4–32.5)	0.7727
Primary disease (RC: NET/UC/FAP)	45:9	66:13	0.9743
Neoadjuvant therapy (done: none)	6:48	24:55	0.0109
Clinical stage (0–II: III)	41:13	47:32	0.0466
PS(0–1: 2–4)	54:0	78:1	0.3062

※ median

*RC* Rectal cancer, *NET* Neuroendocrine tumor, *UC* Ulcerative colitis, *FAP* Familial adenomatous polyposis, *BMI* Body Mass Index, *PS* Performance Status

**Table 2:** Perioperative comparison between the oral inferior group and the oral superior group

	OI (*n*=54)	OS (*n*=79)	*P* value
Surgical procedure (rectal resection: total colectomy)	52:2	75:4	1.0000
Use of anti-adhesion material (use/not use)	2:52	23:56	0.0002
Operation time(minute)※	300.5 (185–475)	297.0 (162–555)	0.7015
Blood loss(ml)※	130 (0–946)	60.0 (0–752)	0.0122
Postoperative hospital stay(day)※	14 (7–49)	16 (9–130)	0.9633
Period until stoma closure(day)※	95 (18–569)	93 (25–560)	0.8177

※ median

*OI* oral inferior; *OS* oral superior

**Table 3:** Postoperative comparison of outlet obstruction, stoma-related complications, intra-abdominal adhesions, thickness of rectus abdominis muscle thickness (horizontal and vertical), length of the straight line connecting both ends of the rectus abdominis muscle, position where the stoma limb penetrated the rectus abdominis muscle, and the angle formed by the rectus abdominis muscle and stoma limbs, between the oral inferior group and the oral superior group

	OI	OS	*P* value
Outlet obstruction	8/54 (14.8%)	1/79 (1.3%)	0.0032
Stoma-related complications (excluded ileus and outlet obstruction)	8/54 (14.8%)	11/79 (13.9%)	1.0000
Degree of intra-abdominal adhesions (none/mild: moderate/severe)	48: 6	61: 18	0.1092
Rectus abdominis muscle thickness (horizontal) (mm) (<10mm: ≥10mm)	23: 31	34: 45	0.9593
Rectus abdominis muscle thickness (vertical) (mm) (<10mm: ≥10mm)	25: 29	42: 37	0.4366
Length of the straight line connecting both ends of the rectus abdominis muscle(mm) ※	64.5 (41.8–95)	64.7 (47.7–93.2)	0.9408
The position where the stoma limb penetrate the rectus abdominis muscle(center-inside: outer)	21: 5	33: 5	0.5187
The angle formed by the rectus abdominis muscle and the stoma limbs(°)※	88.1 (49.6–123.9)	95.95 (74.1–124.1)	0.0161

※ median

The position where the stoma limb penetrated the rectus abdominis muscle, and the angle formed by the rectus abdominis muscle and the stoma limbs were measured in the OI group (26 patients) and the OS group (38 patients)

*OI* oral inferior; *OO* outlet obstruction; *OS* oral superior

**Table 4:** Multivariate analysis conducted using logistic regression analysis revealed that only the oral superior position reduced the incidence of outlet obstruction

	Odds ratio(95%CI)	*P* value
Oral superior	0.1057 (0.0055–0.6294)	0.0106
Blood loss	1.0026 (0.9993–1.0060)	0.1069
Past history of laparotomy	0.3358 (0.0165–2.2494)	0.2907
Use of anti-adhesion material	3.4764e-8 (0–5.9757)	0.3361
Clinical stage	0.5907 (0.0776–2.9606)	0.5414

*OO* outlet obstruction

## Discussion

A diverting stoma is constructed to prevent postoperative leakage after rectal cancer surgery [1, 2, 29]. No consensus exists regarding whether ileostomy or colostomy is better, but ileostomy is often selected in Japan [3–7]. In our department, we tend to perform loop ileostomy because it is easy to construct and close. Ileostomy is a common procedure, but surgeons should be aware of stoma-related complications. Examples of stoma-related complications are skin disorders; stoma prolapse, stenosis, or depression; and parastoma hernia. Bowel obstruction is an important complication often associated with ileostomy [4, 8–10, 30, 31]. OO, which is a type of bowel obstruction, shows stricture only in the stoma part. It can delay the resumption of food intake and also the start of adjuvant chemotherapy, and it carries a risk of intestinal injury from the insertion of an intestinal catheter for treatment. Therefore, clinicians may be forced to perform early stoma closure [15, 32]. OO was reported previously as ileus caused by postoperative ileostomy for UC and FAP [11–13]. Anus-preserving surgery and laparoscopic surgery for lower rectal cancer has recently become popular with increasing opportunities to construct a DLI. The number of reports of OO is increasing; however, clinicians are not fully aware of OO as a complication of DLI [14, 20, 32, 33]. Taking steps to prevent OO is important when surgeons construct and manage a stoma. Several recent reports [18, 34] satisfy conditions 1 and 2 or 1 and 3, which are defined as follows: (1) symptoms of bowel obstruction/ileus symptoms; (2) relief of bowel obstruction by inserting a decompression catheter trans-stomally; and (3) CT findings of a caliber change in the abdominal wall-penetrating part of the stoma but no other obstruction.

The cause of OO is still unclear, but bowel obstruction and OO are more likely to occur with ileostomy than with colostomy [34]. Moreover, intestinal pressure is lower than colonic pressure, which may result in OO, resulting from physical adhesion or relative stenosis at the stoma tunnel by the rectus abdominis muscle [4, 12, 15, 26, 35]. Studies [8–10, 12, 18–20, 27] have also implicated the fascia incision method, mesenteric twist and orientation of the proximal limb, the size of stoma tunnel, and laparoscopic surgery.

As a cause of stenosis related to the rectus abdominis muscle in the stoma tunnel, investigators have mentioned a rectus abdominis muscle thickness of 10 mm or greater, penetration of the center of the rectus abdominis muscle, a sharp angle between the stoma limbs and the rectus abdominis, and laparoscopic surgery [26, 27]. These findings are attributed to the fact that the stoma limbs are longer between the rectus abdominal muscles and are easily tightened by the rectus abdominal muscles [26, 27]. In this study, no significant difference was found between the OI group and the OS group in the thickness and penetration position of the rectus abdominis muscle. Although the angle between the stoma limbs and the rectus abdominis was significantly higher in the OS group, there were only 26 and 38 patients in the OI and OS groups, respectively.

Adhesion of the proximal limb and mesentery to the abdominal wall may be another cause. A study on the fasciotomy method, which compared the vertical incision and the cruciate incision, demonstrated that the cruciate incision results in a larger stoma tunnel [18]. However, OO may occur because of adhesion and bending of the proximal limb at the cruciate incision [18]. We encountered no such cases in this series because all patients had a vertical incision. The use of anti-adhesion agents such as Seprafilm [Baxter International Inc.]) was significantly higher in the OS group, but there was no significant difference between the groups in the degree of intra-abdominal adhesions checked at the time of stoma closure [35].

The size of the stoma tunnel is generally sufficiently large to allow two lateral fingers to pass, although individual differences exist in the size of the two lateral fingers [8–10, 12, 18, 27]. Moreover, muscle relaxation occurs during surgery and as laparoscopic surgery is performed under pneumoperitoneal conditions, these features tend to be risk factors. In the present study, open surgery was performed in both the OI and OS groups and OO rarely occurred; therefore, constructing a sufficient stoma tunnel is important so that the stoma limbs can be passed through with a margin to prevent postoperative stenosis of the stoma tunnel.

When constructing loop ileostomy, the oral side is often constructed at 6 o’clock (OI) to prevent the inflow of stool into the anal side and to facilitate self-care [18, 21, 27]. However, reports indicate that OO is reduced by constructing the oral side at 3 o’clock to induce mesenteric twist [18, 19], although when we observed the inside of the abdominal cavity after pulling the ileum through to construct a DLI during laparoscopic surgery, the mesenteric twist appeared not to be very strong, despite the direction of the proximal limb. We believe that ensuring the stoma limbs do not bend or have stenosis where they penetrate the abdominal wall is important for the prevention of OO.

**Fig. 3:**
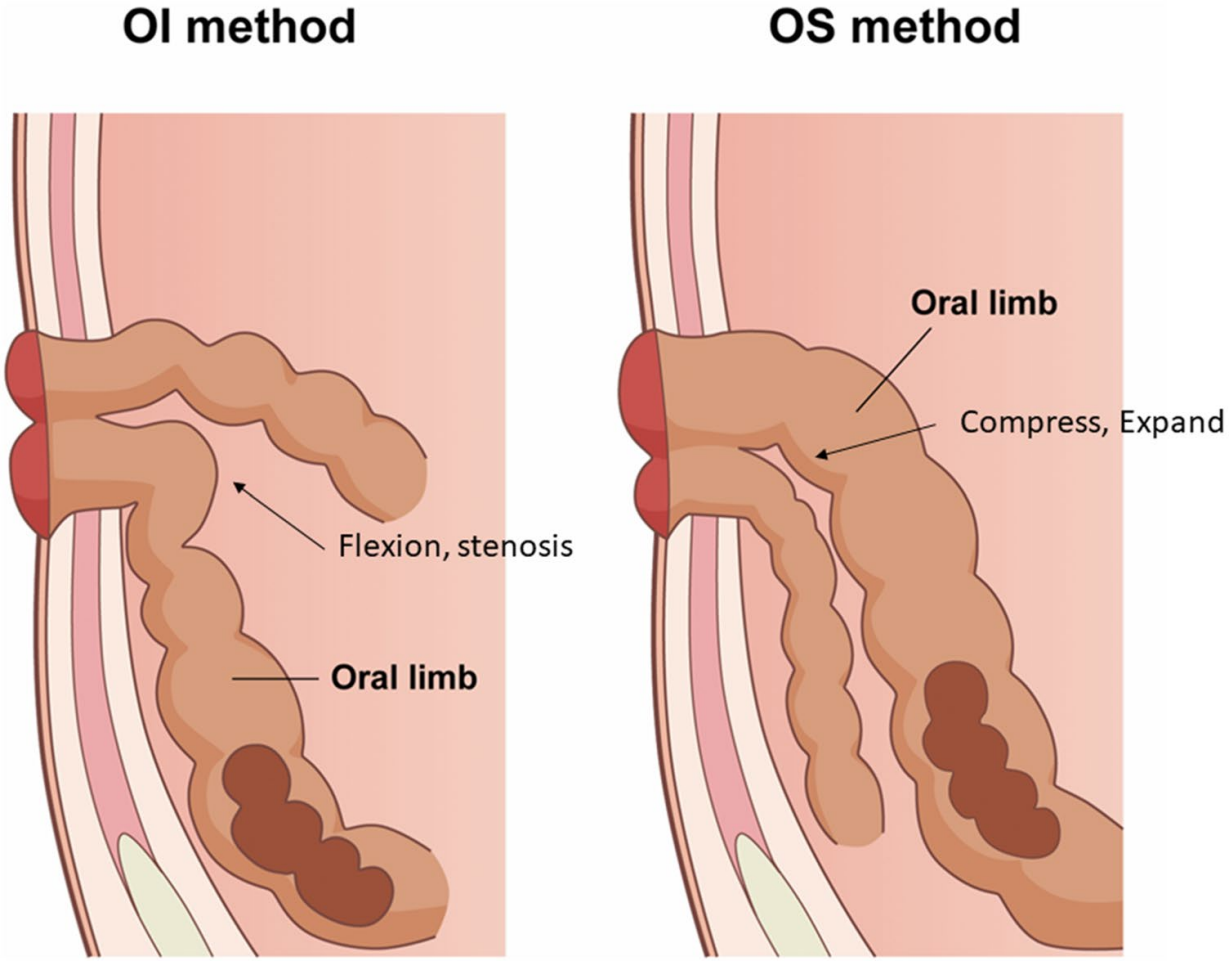
Schema of the stoma limbs in the oral superior method and the oral inferior method with the patient in an upright position

We speculate that the low incidence of OO in our OS group can be explained as follows: As the oral side intestine of the DLI falls into the pelvic cavity postoperatively when the patient is in an upright position, the pooling of stool in the oral side of the intestine results in further gravitational pull toward the caudal side of the proximal limb. Thus, in the OI group, the proximal limb tended to bend where it transitions from the stoma tunnel into the abdominal cavity. Conversely, in the OS group, the anal limb is more compressed if the oral side is pulled more in the caudal direction (Fig. 3, schema). Thus, the proximal limb seems to be able to use the size of the stoma tunnel more effectively and is less likely to narrow.

As the rectus abdominis muscle thickens, the angle from the stoma tunnel to the abdominal cavity becomes steeper. We believe this factor is consistent with previously reported risk factors [26, 27].

Few reports mention the orientation of the proximal limb among the research factors, with no significant difference observed, owing to the small number of cases [20, 26]. Moreover, with regard to DLI, no study has examined whether the proximal limb was oriented to the 12 o’clock position (OS) to prevent OO.

A limitation of this study is that the type of stoma (ileostomy or colostomy) and stoma construction method (OI or OS) may differ, depending on the operator. Moreover, the construction may have been affected by individual differences in the amount of food, digestive and absorptive capacity, and activity and there were possible individual differences in the size of the stoma tunnel (approximately two lateral fingers) in each patient. As OO may still have unknown pathologies, additional prospective studies using a large sample size are needed. However, we believe that the oral superior DLI was effective in preventing postoperative OO.

## Conclusion

This study demonstrated that orienting the proximal limb at the OS position (12 o’clock) when constructing a DLI was a significant factor in preventing OO.
